# 

**Published:** 1872-02

**Authors:** 


### BOOKS RECEIVED.

We have received from Messrs. D. Appleton & Company, 549 and 551 Broadway, New York, the following medical works, which will be carefully noticed in the next number of the Journal:

"Anæsthesia, Hospitalism, Hermaphroditism, and a Proposal to Stamp out Small-Pox and Other Contagious Diseases. By Sir James Y. Simpson, Bart, M.D., D.C.L., late Professor in the University of Edinburgh."

"Hand-Book of Skin Diseases. By Dr. Isidor Neumann, Docent An Der K.K. Universat in Wien. Translated from the Second German Edition, with Notes by Lucien D. Bulkley, A.M., M.D., Surgeon to the New York Dispensary, Department of Venereal and Skin Diseases, etc. Illustrated with Sixty-six Wood-Cuts."

"Treatment of Pulmonary Consumption by Hygiene, Climate and Medicine, in its Connection with Modern Doctrines. By James Henry Bennett, M.D., Member of the Royal College of Physicians, London, etc., etc."

